# The effects of vitamin D on cardiovascular damage induced by lipopolysaccharides in rats

**DOI:** 10.34172/jcvtr.2023.31719

**Published:** 2023-06-29

**Authors:** Narges Marefati, Farimah Beheshti, Akbar Anaeigoudari, Fatemeh Alipour, Reyhaneh Shafieian, Fatemeh Akbari, Maryam Pirasteh, Maryam Mahmoudabady, Hossein Salmani, Sara Mawdodi, Mahmoud Hosseini

**Affiliations:** ^1^Department of Physiology and Medical Physics, Faculty of Medicine, Baqiyatallah University of Medical Sciences, Tehran, Iran; ^2^Applied Biomedical Research Center, Mashhad University of Medical Sciences, Mashhad, Iran; ^3^Neuroscience Research Center, Torbat Heydariyeh University of Medical Sciences, Torbat Heydariyeh, Iran; ^4^Department of Physiology, School of Medicine, Torbat Heydariyeh University of Medical Sciences, Torbat Heydariyeh, Iran; ^5^Department of Physiology, School of Medicine, Jiroft University of Medical Sciences, Jiroft, Iran; ^6^Department of Anatomy and Cell Biology, School of Medicine, Mashhad University of Medical Sciences, Mashhad, Iran; ^7^Department of Physiology, School of Medicine, Mashhad University of Medical Sciences, Mashhad, Iran; ^8^Neuroscience Research Center, Mashhad University of Medical Sciences, Mashhad, Iran; ^9^Student Research Committee, Jiroft University of Medical Sciences, Jiroft, Iran; ^10^Pharmacological Research Center of Medicinal Plants, Mashhad University of Medical Sciences, Mashhad, Iran; ^11^Psychiatry and Behavioral Sciences Research Center, Mashhad University of Medical Sciences, Mashhad, Iran

**Keywords:** Lipopolysaccharide, Cardiovascular, Vitamin D, Oxidative Stress, Inflammation, Fibrosis

## Abstract

**Introduction:**

Inflammation and oxidative stress are contributed to cardiovascular diseases. Vitamin D (Vit D) has antioxidant and anti-inflammatory properties. In the current research, the effect of Vit D on cardiac fibrosis and inflammation, and oxidative stress indicators in cardiovascular tissues was studied in lipopolysaccharides(LPS) injected rats.

**Methods:**

Rats were distributed into 5 groups and were treated for 2 weeks. Control: received vehicle(saline supplemented with tween-80) instead of Vit D and saline instead of LPS, LPS: treated by 1 mg/kg of LPS and was given vehicle instead of Vit D, LPS-Vit D groups: received 3 doses of Vit D (100, 1000, and 10000 IU/kg) of Vit D in addition to LPS. Vit D was dissolved in saline supplemented with tween-80 (final concentration 0.1%) and LPS was dissolved in saline. The white blood cell (WBC) was counted. Oxidative stress markers were determined in serum, aorta, and heart. Cardiac tissue fibrosis was also estimated using Masson’s trichrome staining method.

**Results:**

WBC and malondialdehyde (MDA) were higher in the LPS group than the control group, whereas the thiol content, superoxide dismutase (SOD), and catalase (CAT) were lower in the LPS group than the control group (*P*<0.01 and *P*<0.001). Administration of Vit D decreased WBC (*P*<0.001) and MDA (*P*<0.05 and *P*<0.001) while enhanced thiol (dose 10000 IU/Kg) (*P*<0.001), SOD (dose 10000 IU/kg) (*P*<0.001), and CAT (*P*<0.05 and *P*<0.001) compared to the LPS group. All doses of Vit D also decreased cardiac fibrosis compared to the LPS group (*P*<0.001).

**Conclusion:**

Vit D protected the cardiovascular against the detrimental effect of LPS. This cardiovascular protection can be attributed to the antioxidant and anti-inflammatory properties of Vit D.

## Introduction

Oxidative stress is an outcome of over-generation or disturbance in the neutralization of reactive oxygen species (ROS).^1^ ROS and oxidative stress have an important contribution to the induction and development of cardiovascular diseases (CVD).^2^ The most well-known ROS causing cardiovascular disturbances are hydrogen peroxidase, nitric oxide, and superoxide anion.^3^ Over-release of these free radicals from intracellular different sources has been reported to alter the normal function of endothelial cells and cardiac myocytes.^4^ Oxidative stress has been also shown to be a causative factor in the induction of cardiac hypertrophy and heart fibrosis resulting from long-term hypertension.^5^

Lipopolysaccharide (LPS) in the cell wall of bacteria is one of the most powerful stimuli in the production and release of ROS.^6^ The LPS-linked endothelial malfunction has been understood to be a critical factor in the pathogenesis of CVD.^7^ The binding of LPS to its receptor, toll-like receptor 4 (TLR4), triggers a cascade of intracellular signaling events causing endothelial damage.^8^ Using in vivo studies it has been shown that LPS administration has been also shown to induce oxidative stress in the cardiovascular tissues of rats.^9-11^ Alongside the induction of oxidative stress, LPS also stimulates the immune cells to release inflammatory mediators.^12^ One of the principal intracellular signaling pathways targeted by LPS to induce immune reactions is the nuclear factor κB (NFκB) pathway.^13^

Vitamin D (Vit D) is a steroid molecule that has several physiological effects including enhancement of calcium absorption from the intestine and increase of calcium deposition in bone tissue.^14^ The role of this fat-soluble vitamin in the modulation of oxidative stress status in various tissues has been also demonstrated.^15^ For example, Vit D has been revealed to alleviate retinopathy induced by oxidative stress in diabetic rats.^16^ It has been also corroborated that lack of Vit D in rats led to endothelial injury accompanied by nitrosative stress.^17^ In a genomic manner, Vit D also inhibits the transcription from the gene of NFκB and tumor necrosis factor-α (TNF- α) receptor, therefore suppressing inflammatory responses.^18^ In the present research, we decided to find additional evidence on the effect of Vit D on cardiac tissue fibrosis and oxidative stress indicators in cardiovascular tissues in LPS-injected rats.

## Materials and Methods

###  Animals and groups

In this study, the experiments were conducted on 35 adult male Wistar rats ( weighing 203-250 g and 10-12 weeks old). G power software was used to calculate the sample size. The animals enjoyed from a room with standard conditions (cycle of 12 light/dark, appropriate temperature 22 ± 2 ^◦^C, and adequate amount of food and water). Rats were randomly assigned to the following groups(n = 7 in each group): 1. Control: received vehicle (saline supplemented with tween-80, 2 ml/kg) instead of Vit D and saline (1ml/kg) instead of LPS, 2. LPS: received vehicle (2ml/kg) instead of Vit D and treated with 1 mg/kg of LPS, 3. LPS-Vit D100: treated with 100 IU/kg of Vit D plus 1 mg/kg of LPS, 4. LPS-Vit D1000: received 1000 IU/kg of Vit D plus 1 mg/kg of LPS, 5. LPS-Vit D10000: received 10000 IU/Kg of Vit D plus 1 mg/kg of LPS. Vit D was dissolved in saline supplemented with tween-80 (final concentration 0.1%) and LPS was dissolved in saline. Injections including saline, Vit D, and LPS were accomplished intraperitoneally (IP) for 2 weeks. The type of Vit D used in this study was D3(cholecalciferol) and it was purchased from the Darupakhsh Pharmaceutical Company, Iran (Catalog NO: 34443). The previous studies were considered and the doses of Vit D were chosen.^19-21^ In previous studies, it was shown that these doses were safe without mortality.^19-21^ After 2 weeks, the rats were deeply anesthetized using high doses of ketamine and xylazine, the chest was opened, and the blood samples were collected by cardiac puncture. To do this, the heart was pierced with a 4-gauge needle that was inserted into the heart and systemic blood was drained into the syringe. No serious mechanical damage was done to the heart, and the blood sampling method was applied in the same way in all groups. The blood samples were dispensed into the tubes(Greiner Company, Germany) containing EDTA (ethylenediaminetetraacetic acid) to be used for determining white blood cells (WBC). Also, 2 ml of the blood samples were centrifuged, the serum samples were separated and finally were used for measurement of oxidative stress indicators. In addition, the heart and aorta tissues were separated and kept for later use. The level of malondialdehyde (MDA) and thiol groups, as well as catalase (CAT) and superoxide dismutase (SOD) activity, were evaluated in serum, heart, and aorta. The left ventricls were used to be stained with Masson’s trichrome and analyzed under light microscopy for cardiac fibrosis. All institutional and national guidelines for the care and use of laboratory animals were followed. The procedures were approved by the Ethics Committee of Animal Studies at Mashhad University of Medical Sciences (IR.MUMS.MEDICAL.REC.1399.301 ).

###  Measurement of WBC 

 The WBC was counted using an automated hematologic analyzer( Hitachi, Japan) in a medical laboratory( Navid).

###  Evaluation of oxidative stress criteria

The oxidative stressmarkersweredetermined in serum and homogenized tissue of the aorta and heart. The method was as previously reported.^22,23^ The level of MDA was appraised in the presence of trichloroacetic acid (TBA, Sigma Aldrich Company, United States, Catalog NO: T5500) as a reagent. After adding TBA, samples were heated for 30 min. The red complex resulting from the reaction of MDA with TBA has an absorbance peak at 532 nm.^19^

Determination of the level of thiol groups was carried out using 2-nitrobenzoic acid (DTNB, Sigma Aldrich Company, United States, Catalog NO: 103291). In this colorimetric method, a yellow compound with an absorbance peak of 412 nm appeared when DTNB reacted with thiol groups. The method was as previously reported.^19,22,23^ The activity of SOD was measured based on pyrogallol auto-oxidation for the formation of superoxide. Superoxide then converts tetrazolium into red formazan with absorbance at 560 nm. The method was as previously described.^20^

In employing Aebi manner to determine the CAT activity, hydrogen peroxide was dissociated into water and oxygen in the presence of CAT. A high level of CAT resulted in a decline in absorbance.^20^

###  Histological method

For histology assay, hearts were removed and the left ventricles were harvested and kept in 10% formalin for 48 hours to be fixed. After paraffin embedding, and cutting into 5 μm sections. The sections were deparaffinized in xylene, rehydrated in graded alcohol, and transferred to 0.01 M phosphate-buffered saline (PBS, pH 7.4). The sections were then stained using Masson’s trichrome technique according to the guideline of the agent kit. A light microscopy technique was used to analyze the tissues for fibrosis. In this technique, cardiomyocytes and other elements in the background except collagen fibers stain red, collagen fibers blue, and nuclei black. The area of collagen deposition (blue-stained) and extended quantity of connective tissue around the vessels was assigned as fibrosis. Briefly, the slides were placed in Bouin’s solution for 1 hour in a 55 -56 degree oven, and rinsed in distilled water for 3 min. After incubation in Weigert’s Iron Hematoxylin solution (staining the nuclei in black) for 15 minutes, washed in distilled water. Then the slides were submerged in 1% Biebrich Scarlet-Acid Fuchsin (staining the background in red) for 1 minute, then washed in distilled water for 5 min. After differentiation in phosphomolybdic-phosphotungstic acid solutions for 15 min, sections were transferred directly into aniline blue solution (staining the collagen fibers in blue) for 15 min. Heart samples were then differentiated in 1% acetic acid solution for 3 min, dehydrated in 95 and 100% ethanol, then cleared in xylene, and mounted with a coverslip. Image J software was applied to analyze average staining intensity to determine fibrosis in the heart. This software is utilized for staining qualitative data. After acquiring the staining intensity by this software, the average staining intensity was converted to an optical density (OD) using the following formula for more statistical analysis. In this formula, the max intensity is 255 as a fixed value.^24^


$$Optical\;Density=Log\left(\frac{Max\;intensity}{Mean\;intensity}\right)$$

###  Statistical analysis 

Findings were analyzed by analysis of variance (one-way ANOVA). Tukey post hoc test was used for the comparison of the groups. Differences were significant when *P* < 0.05.

## Results

###  The results of the WBC count

**Figure 1:**
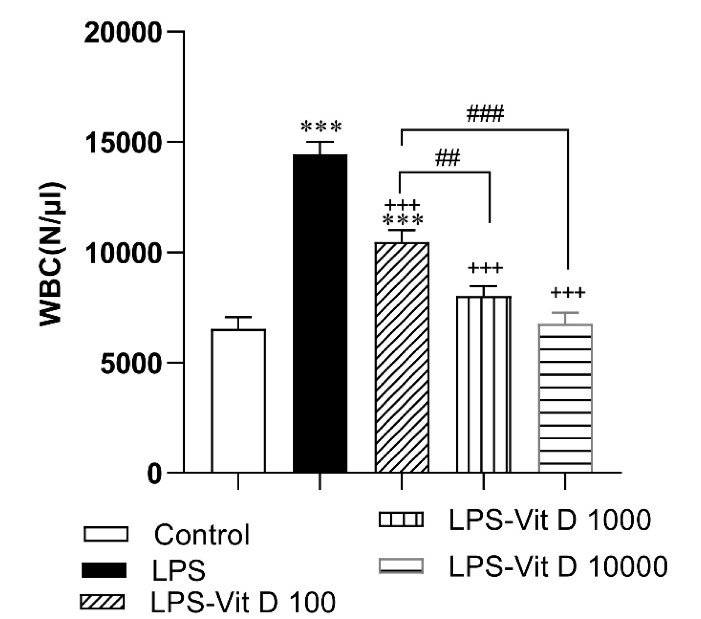
Comparison of the number of white blood cells (WBC) among different groups. Data was expressed as Mean ± SEM.***P < 0.001 compared to the control group, ^+ + +^*P* < 0.001 compared to the LPS group, ^##^*P* < 0.01 and ^###^*P* < 0.001 compared to the LPS-Vit D 100 group

As shown in Figure 1, LPS elevated the number of WBC in the blood samples of the rats of the LPS group compared with the control group (*P* < 0.001). Administration with all three doses of Vit D considerably lowered WBC in LPS-Vit D groups with respect to the LPS group (*P* < 0.001). In addition, the decrease of WBC in the LPS-Vit D1000 and LPS-Vit D10000 groups was more than in the LPS-Vit D100 group (*P* < 0.01 and *P* < 0.001)(Figure 1).

###  Oxidative stress indicators

**Figure 2:**
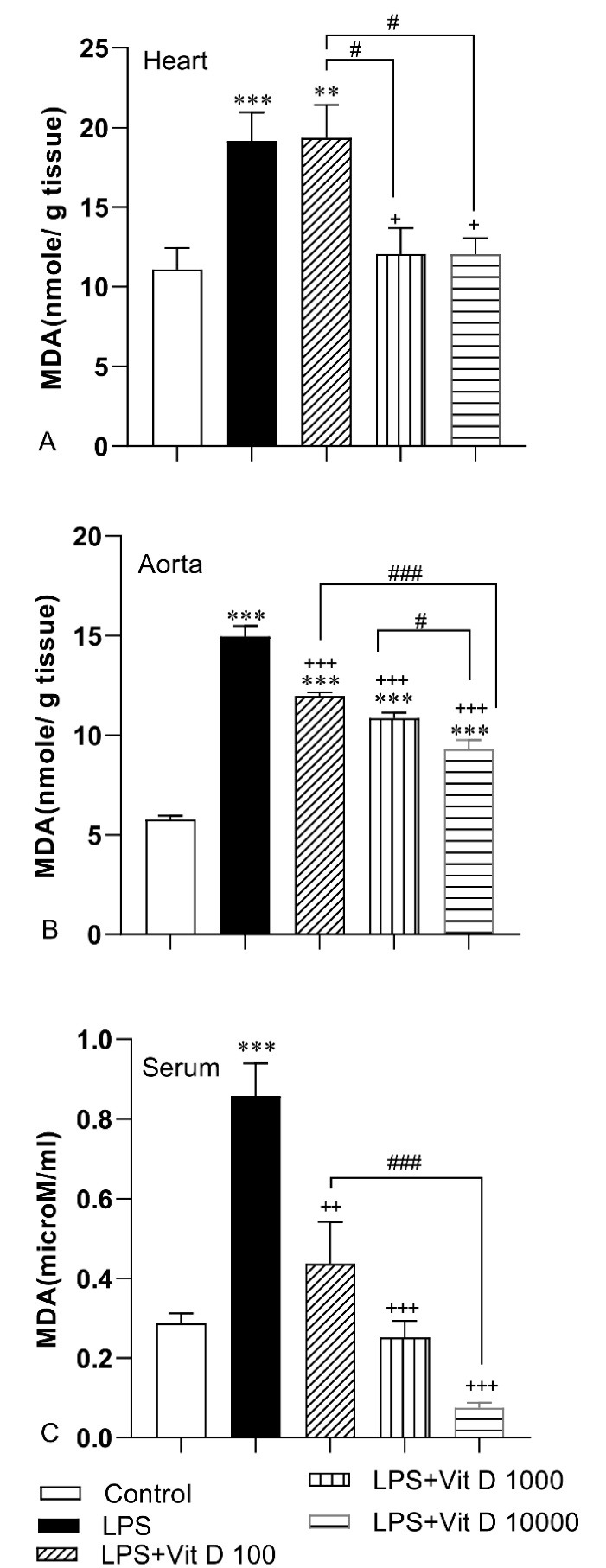
Comparison of malondialdehyde (MDA) concentration in heart (A), aorta (B) and serum(C) among different groups. Data was expressed as Mean ± SEM. ** *P* < 0.01 and***P < 0.001 compared to the control group, ^+^*P* < 0.05, ^+ +^*P* < 0.01 and ^+ + +^*P* < 0.001 compared to the LPS group, ^#^*P* < 0.05, ^##^*P* < 0.01 and ^###^*P* < 0.001 compared to other doses of Vit D group

It is deduced from Figure 2 that the accumulation of MDA happened in heart and aorta tissues and serum following LPS injection in the LPS group compared to the control group (*P* < 0.001). The results of Figure 2A indicate that there was no noticeable difference in the MDA concentration of heart tissue in the LPS-Vit D100 group with respect to the LPS group. Vit D administration with 1000 and 10000 doses could decrease significantly the level of MDA in heart tissue of LPS-Vit D1000 and LPS-Vit D10000 groups than the LPS group (*P* < 0.05). Data analysis also demonstrated that all three doses of Vit D could mitigate remarkably the level of MDA in aorta tissue and serum of LPS-Vit D groups compared LPS group (*P* < 0.001). Per the results, the accumulation of MDA in heart and aorta tissues and serum of LPS-Vit D10000 was lower than LPS-Vit D100 group (*P* < 0.05 to *P* < 0.001). In addition, MDA concentration in the heart of the LPS-Vit D1000 group was lower than the LPS-Vit D100 group(*P* < 0.05). Finally, the MDA level in the aorta of the LPS-Vit D10000 group was lower than the LPS-Vit D1000 group(*P* < 0.05).

**Figure 3:**
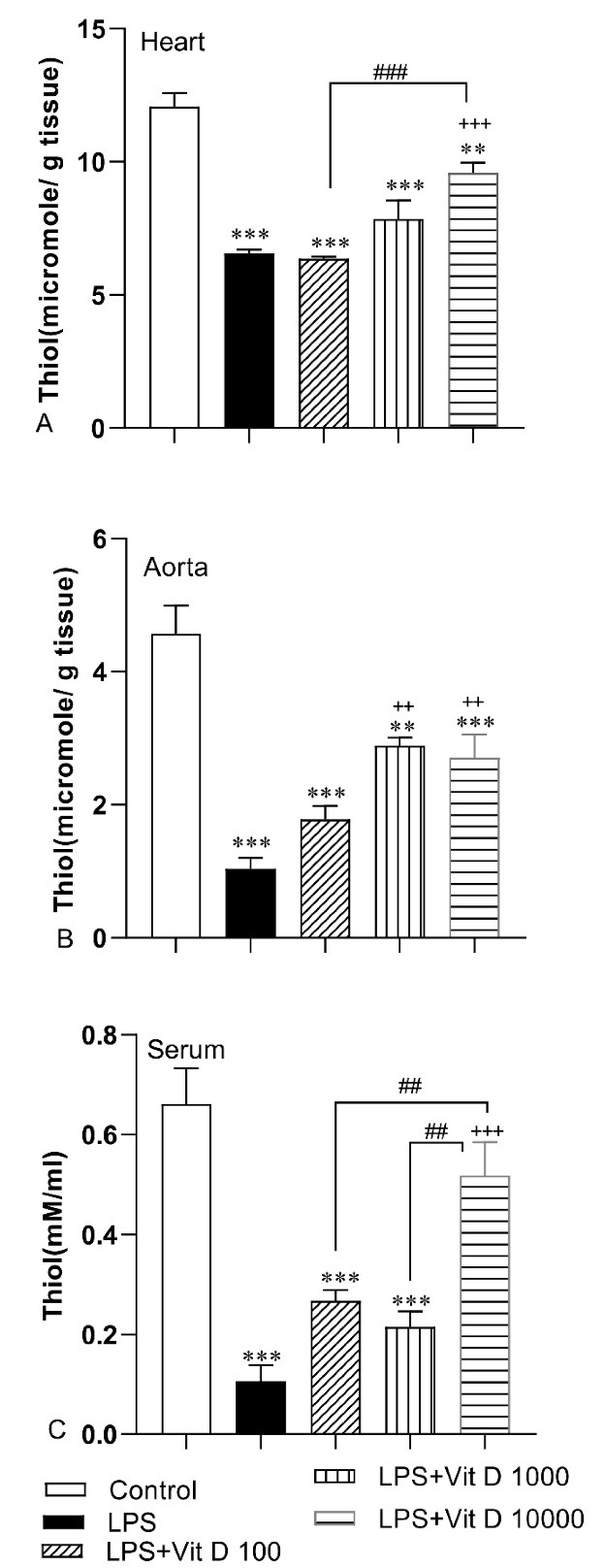
Comparison of total thiol concentration in heart (A), aorta (B) and serum(C) among different groups. Data was expressed as Mean ± SEM. ** *P* < 0.01 and ***P < 0.001 compared to the control group, ^+ +^*P* < 0.01 and ^+ + +^*P* < 0.001 compared to the LPS group, ^##^*P* < 0.01 and ^###^*P* < 0.001 compared to other doses of Vit D

The total thiol concentration in all heart, aorta, and serum in LPS-treated rats was lower than those of the control group (*P* < 0.001). The content of this antioxidant parameter was enhanced in heart tissue and serum of rats treated with 10000 IU/kg of Vit D when it was compared with the LPS group (*P* < 0.001). The concentration of total thiol groups in heart tissue and serum of LPS-Vit D100 and LPS-Vit D1000 groups had no significant difference with the LPS group. The amount of total thiol in the aorta tissue of LPS-Vit D1000 and LPS-Vit D10000 groups was higher than LPS groups (*P* < 0.01). There was not any difference in the concentration of total thiol in aorta tissue in the LPS-Vit D100 group compared with the LPS group. In addition, the total thiol concentration in the heart and serum of the LPS-Vit D10000 group was higher than the LPS-Vit D100 group (P < 0.001 and P < 0.01). Finally, the serum thiol content in the LPS-Vit D10000 group was higher than the LPS-Vit D1000 group (*P* < 0.01) (Figure 3A, 3B, and 3C).

**Figure 4:**
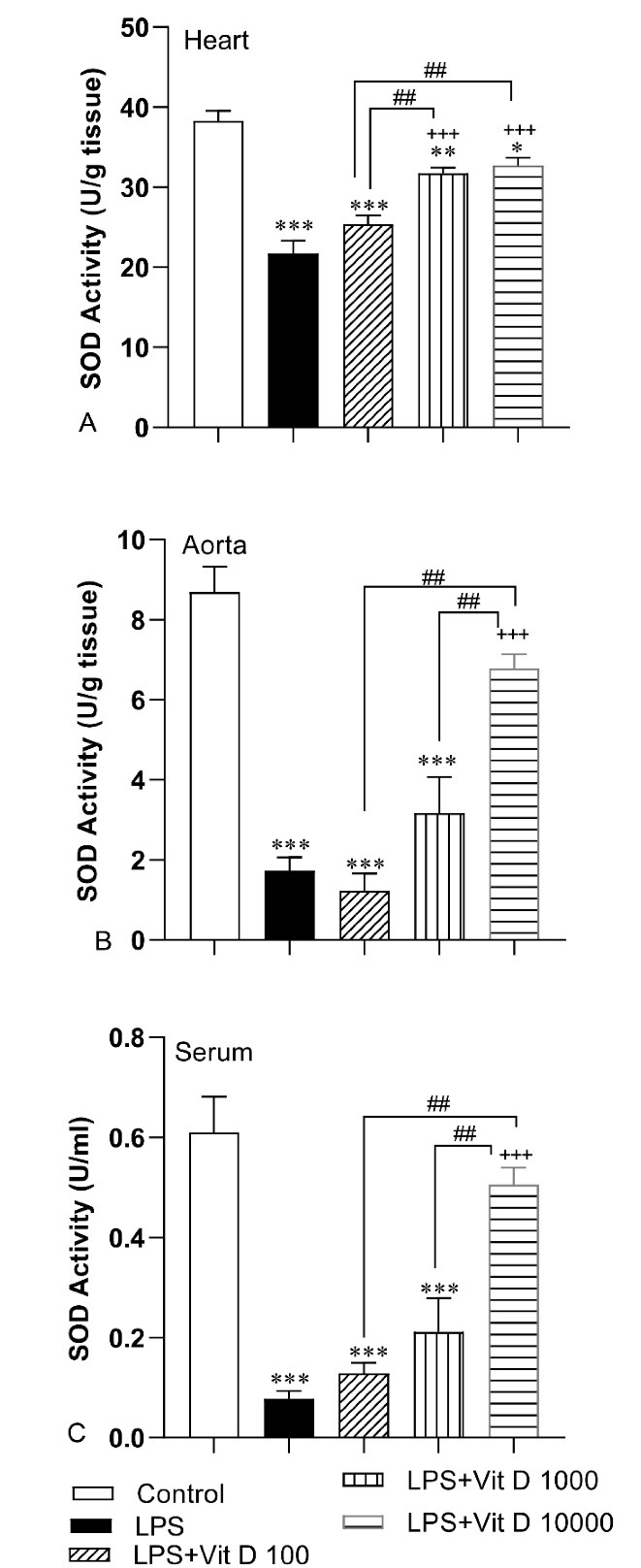
Comparison of superoxide dismutase (SOD) activity in heart (A), aorta (B) and serum(C) among different groups. Data was expressed as Mean ± SEM. * *P* < 0.05, ** *P* < 0.01 and ***P < 0.001 compared to the control group, ^+ + +^*P*< 0.001 compared to the LPS group, ^##^*P* < 0.01 and ^###^*P* < 0.001 compared to other doses of Vit D

According to Figure 4, a decreased activity of SOD occurred in the cardiovascular system of the animals of the LPS group with respect to the control group (*P* < 0.001). Injection of 1000 and 10000 IU/kg of Vit D elevated the SOD activity in heart tissue of LPS-Vit D1000 and LPS-Vit D10000 groups compared with the LPS group (*P* < 0.001). Based on the findings, we did not behold noticeable significance in SOD activity in the heart tissue of the LPS-Vit D100 group compared with the LPS group. The activity of SOD in the aorta tissue and serum of rats treated with 10000 IU/kg of Vit D was higher than the LPS group (*P* < 0.001). Analysis of data demonstrated that there was no significant difference in SOD activity in aorta tissue and serum of LPS-Vit D100, LPS- Vit1000 groups than LPS group. The SOD activity in the heart and aorta tissue and serum of the LPS-Vit D10000 group and also in the heart of the LPS-Vit D1000 group was higher than the LPS-Vit D100 group (*P* < 0.01 to *P* < 0.001). Comparison results indicated that the activity of SOD in aorta tissue and serum of LPS-Vit D10000 was more than LPS-Vit D1000 (*P* < 0.01).

**Figure 5:**
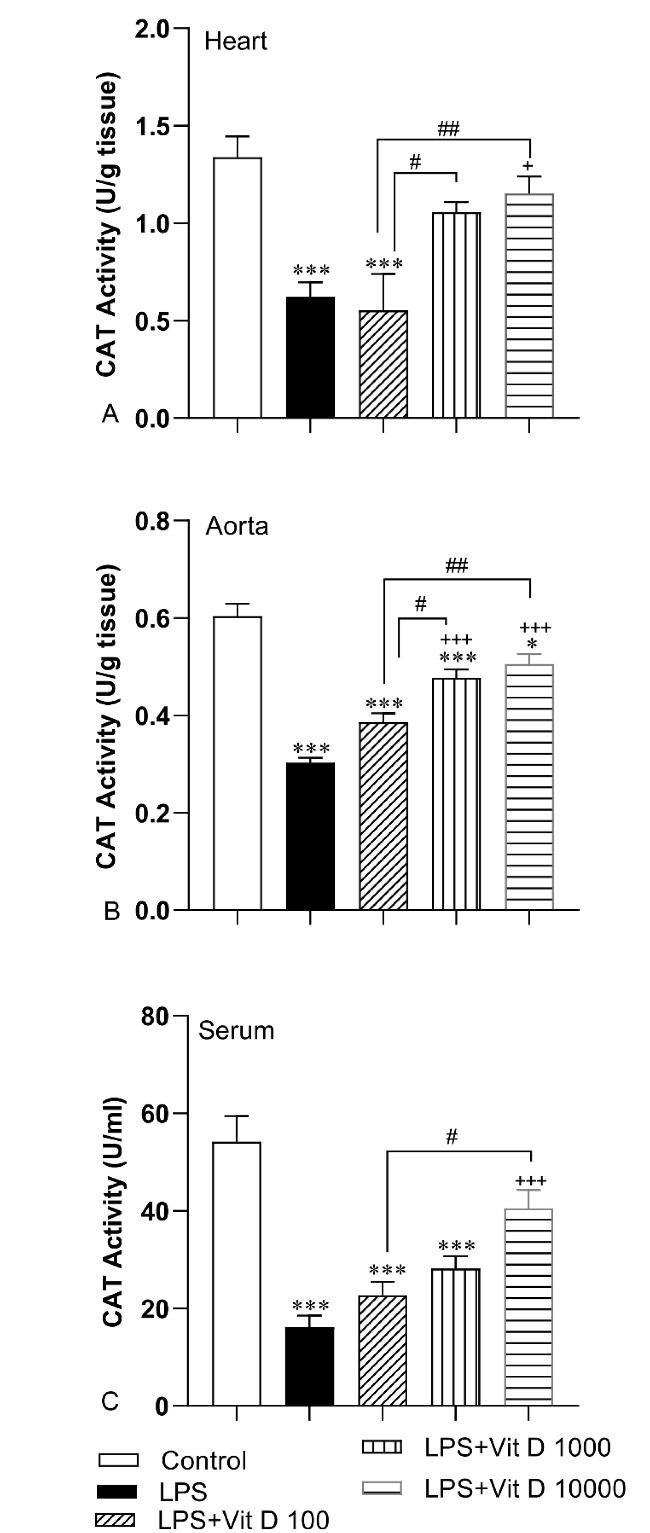
Comparison of catalase(CAT) activity in heart (A), aorta(B) and serum(C) among different groups. Data was expressed as Mean ± SEM. *P < 0.05 and ***P < 0.001 compared to the control group, ^+^*P* < 0.05 and ^+ + +^*P* < 0.001 compared to the LPS group, ^#^*P* < 0.05 and ^##^*P* < 0.01 compared to the LPS-Vit D100 group

In accordance with Figure 5, LPS administration resulted in a significant decrement in CAT activity of the heart and aorta tissue and serum LPS group compared with the control group (*P* < 0.001). Injection of 1000 and 10000 IU/kg of Vit D caused a significant increase in CAT activity in aorta tissue in LPS-Vit D1000 and LPS-Vit D10000 groups compared with the LPS group (*P* < 0.01 and *P* < 0.001). Administration with Vit D also enhanced CAT activity in heart tissue and serum of the LPS-Vit D10000 group with respect to the LPS group (*P* < 0.05 and *P* < 0.001). The activity of CAT in heart tissue and serum in LPS-Vit D100 and LPS-Vit D1000 had no difference from the LPS group. In addition, we did not see any significant difference in the activity of this antioxidant enzyme in the aorta tissue of the LPS-Vit D100 group with respect to the LPS group. The activity of CAT in heart and aorta tissue in LPS-Vit D1000 and LPS-VitD10000 was higher than LPS-Vit D100 (*P* < 0.05 and *P* < 0.01). Additionally, CAT activity in the serum of the LPS-Vit D10000 group was higher than LPS-Vit D100 group (*P* < 0.05) (Figure 5A, 5B and 5C).

###  The results of cardiac fibrosis

**Figure 6:**
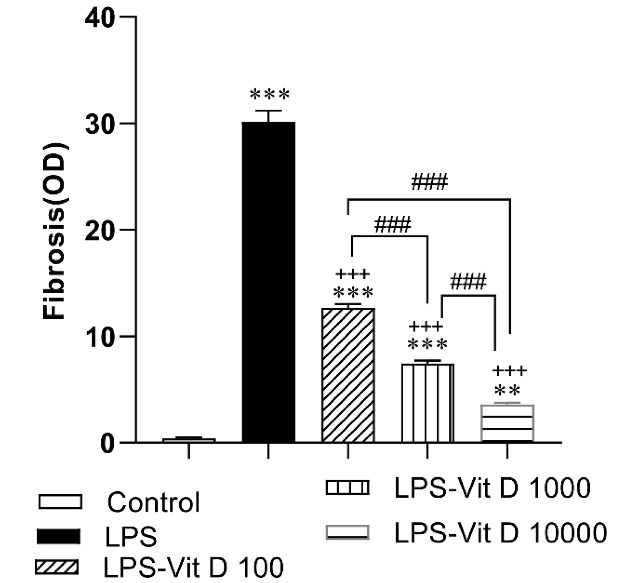
The results of fibrosis in the heart tissue. The data were described as mean ± SEM (n = 7 in each group). ***P < 0.001 compared to the control group, ^+ + +^*P* < 0.001 compared to the LPS group, ^###^*P* < 0.001 compared to other doses of Vit D

**Figure 7:**
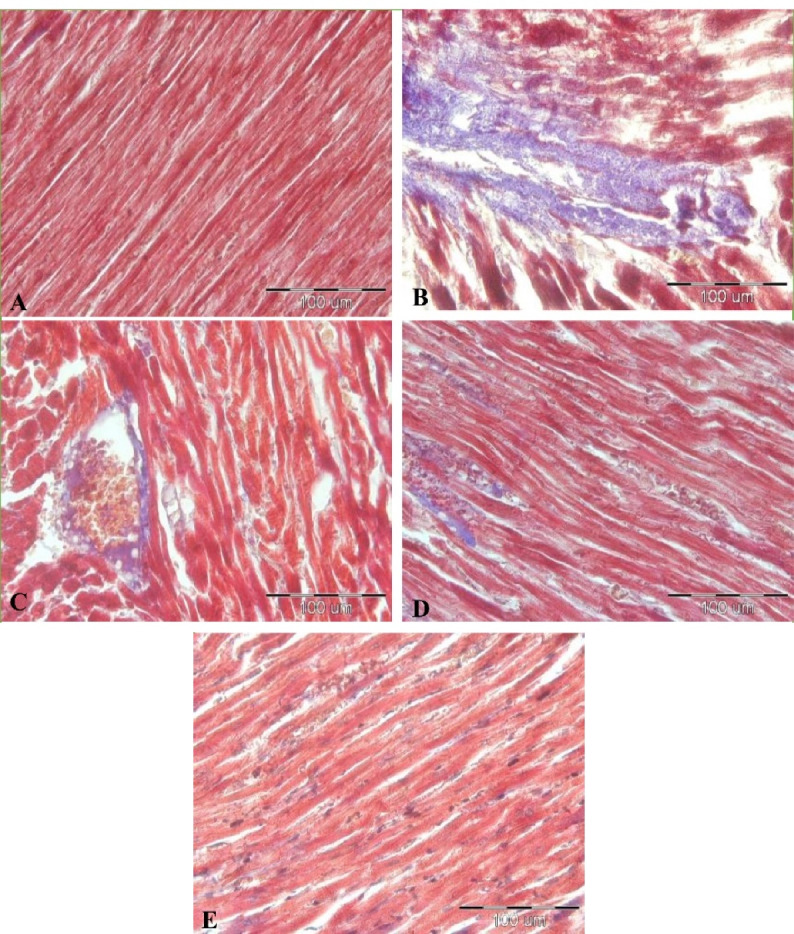
The photos of heart tissue stained with Masson’s Trichrome (400 × magnification). Blue color indicates collagen fibers (yellow arrow). (A) Control, (B) LPS, (C) LPS-Vit D100, (D) LPS-Vit D1000, and (E) LPS-Vit D10000 groups

The results of staining with Masson’s trichrome revealed that there was cardiac fibrosis in all LPS injected groups compared to the control group (*P*< 0.001 all)(Figures 6 and 7). Also, our results showed that treatment by all doses of Vit D decreased cardiac fibrosis in all LPS-Vit D100, LPS-Vit D1000, and LPS-VitD10000 groups compared to the LPS group (*P*< 0.001 all). The effect of Vit D was dose-dependent and cardiac fibrosis in the LPS-VitD10000 group was lower than that in LPS-Vit D100 and LPS-Vit D1000 groups (*P*< 0.001). Cardiac fibrosis in the LPS-Vit D1000 group was lower than that in the LPS-Vit D100 group(*P* < 0.001) (Figures 6 and 7).

## Discussion

 The findings of the present study show that oxidative damage followed by LPS injection in the cardiovascular system of rats was alleviated by Vit D. The results also showed that Vit D attenuated cardiac fibrosis induced by LPS injection.

The previous studies showed that high levels of ROS production and oxidative damage are followed by an increase in muscle mass of the heart and cardiac dysfunction.^24^ The uncontrolled generation of free radicals such as nitric oxide plays a pivotal role in cardiac hypertrophy, hypertension, and heart disorders.^25^ In addition, oxidative stress is followed by an endothelial malfunction that has been visualized to be an important cause of the progression of CVD.^26^ It has also been recognized that endothelial dysfunction resulting from LPS injection contributes to the pathogenesis and development of CVD.^7^ LPS has been found to disturb the normal function of endothelial cells via overexpression of adhesion molecules such as vascular cell adhesion molecule 1(VCAM-1) and P-selectin.^27^

In addition, LPS can enhance the generation of inflammatory cytokines and oxidative stress indicators in vessels and the heart.^6^ One of the known key enzymes in ROS production is NADPH oxidase. Suppression of NADPH oxidase activity has been suggested to improve aorta dysfunction in rats.^28^ It has been also suggested that LPS can promote the expression of pro-inflammatory mediators and free radicals via activating NADPH oxidase.^29^ In line with these reports, the oxidative injury resulting from LPS administration in the cardiovascular system of rats was seen in the present work. In confirmation of this finding, LPS injection caused a noteworthy augmentation in MDA level and considerable depletion in total thiol concentration and the activity of CAT and SOD in the aorta and heart tissues, and the serum in the rats. The results also showed that inflammation and oxidative stress induced by LPS injection were followed by cardiac fibrosis. The results are similar to the previous reports in which LPS administration induces a systemic inflammation status in the rats accompanied by oxidative stress in the cardiovascular system and cardiac fibrosis. ^11,30-32^ Cardiac fibrosis is considered to be a connecting factor between inflammation and cardiovascular dysfunction.^33^

There are various antioxidant agents which can ameliorate CVD.^34^ Vitamins are organic compounds that can exert potent antioxidant effects against cardiovascular disturbances.^35^ Improvement of endothelial dysfunction due to hypertension, smoking, hyperlipidemia, and high blood glucose has been attributed to vitamins with antioxidant effects such as E and C.^36^ In addition, the protective effect of Vit D against oxidative damage has been documented.^37^ Based on previous studies Vit D could attenuate microvascular problems induced by uncontrolled diabetes. These vascular protective effects were associated with a reduction in oxidative stress.^38^ The hepatoprotective effect of Vit D via the reduction of MDA concentration, enhancement of glutathione peroxidase activity, and inhibition of the release of apoptotic factors in a rat model of ischemic/reperfusion has been approved.^39^ It has been also understood that Vit D deficiency led to endothelial malfunction associated with oxidative and nitrosative stress in the aorta of rats.^17^ These findings support the results of our study that Vit D modulated the level of oxidative stress indicators in the aorta and heart tissues as well as serum through the decline of MDA and rise of total thiol group and SOD and CAT activity.

In this study, Vit D lowered the number of WBC in LPS-Vit D groups compared to the LPS group. In various studies, the high level of WBC has been considered as an indicator for severe reactions of the immune system.^40^ LPS has been also introduced as a potent stimulator affecting immune cells.^41^ Previously, LPS use has been found to elevate the level of WBC and augment the inflammatory cytokines.^42^ On the other hand, apart from antioxidant effects, anti-inflammatory properties of Vit D have been shown.^43^ Overexpression of anti-inflammatory mediators including interleukin (IL)-10, inhibition of release of inflammatory cytokines such as IL-12, and suppression of NFκB activity have been attributed to Vit D.^37^ Concerning these findings, it is suggested that inhibition of inflammatory responses by Vit D may have a role in the results of our study however, it can be checked to illuminate in the future. A comparison of the effects of used doses of Vit D on cardiovascular parameters and oxidative stress indexes indicated that a high dose of this fat-soluble vitamin was more effective than the other two doses.

Considering the histological results it seems that Vit D attenuated oxidative stress and inflammation and by which decreased cardiac fibrosis. The effects of Vit D on cardiac fibrosis in inflammation status have not been reported to compare with the results of current research but in a myocardial infarction model, Vit D supplementation improved cardiovascular function and decreased myocardial fibrosis.^44^ The results of clinical studies also suggested that Vit D deficiency contributes to inflammation, remodeling, fibrosis, and atherosclerosis in patients with heart failure.^45^ Vit D deficiency has been reported to be a link between oxidative stress cardiac fibrosis, and increased levels of collagen synthesis, and all these components are considered to be main contributors to cardiovascular diseases including heart failure.^46^ Considering the results of the present study, it seems that Vit D can reduce cardiovascular complications of inflammation however, some more clinical experiments is suggested to be done.

## Conclusion

 Vit D protected the cardiovascular against the detrimental effect of LPS. This cardiovascular protection can be attributed to the antioxidant and anti-inflammatory properties of Vit D. As a limitation of this study, cellular and molecular studies using more precise methods need to be done to investigate the further mechanism(s).

## Acknowledgments

 The work is extracted from aMedical (MD) student thesis supported by the Research Council of Mashhad University of Medical Sciences (No. 990667).

## Competing Interests

 There is no conflict of interest to declare.

## Ethical Approval

 The methods of the manuscript were approved by the Committee on Animal Research of Mashhad University of Medical Sciences (Ethical code: IR.MUMS.MEDICAL.REC.1399.301) on 2020-07-29.

## Funding

 The work was financially supported by the Research Council of Mashhad University of Medical Sciences (No. 990667).
